# Development and feasibility of randomized trial to reduce urinary bisphenols in women with obesity

**DOI:** 10.1186/s40814-020-00744-5

**Published:** 2021-01-12

**Authors:** Todd Hagobian, Zoe Delli-Bovi, Adrian Mercado, Alyssa Bird, Megan Guy, Suzanne Phelan

**Affiliations:** Center for Health Research and Department of Kinesiology and Public Health, California Polytechnic State University, 1 Grand Avenue, San Luis Obispo, CA 93407 USA

**Keywords:** Bisphenol A, Body weight, Diet, Endocrine disruptors

## Abstract

**Background:**

Bisphenol exposure is widespread and correlated with diabetes and cardiovascular disease. Previous intervention studies have successfully lowered bisphenol exposure among women of normal weight. The primary objective of this study was to develop and test the feasibility of a 3-week behavioral change intervention, rooted in social cognitive theory, to lower a broad range of bisphenols (BPA, BPS, and BPF) in women with obesity.

**Methods:**

Thirty women with obesity (31.1 ± 5.6 kg/m^2^, 21.1 ± 3.1 years) were randomly assigned to an intervention or control. The intervention included weekly face-to-face meetings to reduce bisphenol exposures from food, cosmetics, and packaged products. Fasting urinary bisphenols, creatinine, and weight were assessed at study entry and after 3 weeks.

**Results:**

The intervention was evaluated as feasible (100% of enrollment and recruitment, 96% of retention and attendance at lesson plan visits, and 96% of a collection of urine samples). Adherence to the intervention was estimated based on completion of self-monitoring records; the number of daily records completed was 7.7 ± 1.3 (mean ± SD) after week 1, 7.1 ± 1.5 after week 2, and 4.4 ± 0.9 after week 3. In secondary analysis, there was a significant treatment × time effect on creatinine-corrected urinary BPS (− 1.42 μg/g creatinine in the intervention vs. − 0.09 μg/g creatinine in the control group).

**Conclusion:**

In women with obesity, the 3-week intervention was considered feasible with promising preliminary results of decreasing BPS concentrations. These data warrant future large-scale clinical trial interventions to reduce bisphenol exposure and determine whether reductions in bisphenols positively impact diabetes and cardiovascular disease risk markers. This study was retroactively registered at ClinicalTrial.gov Identifier NCT03440307.

## Background

Non-persistent endocrine-disrupting chemicals, including bisphenol A (BPA) and analogs bisphenol S (BPS) and bisphenol F (BPF), are compounds routinely used in the production of plastics, appearing in the lining of food and beverage containers and several other products commonly used by consumers [1–3]. The human exposure to bisphenols is extensive; an analysis of the National Health and Nutrition Examination Survey (NHANES) data showed that 93% of the US population had detectable levels of BPA [4]. Observational data has shown positive associations between urinary BPA concentrations and type-2 diabetes [5], peripheral artery disease [6], metabolic syndrome [7], and obesity [8]. A recent study indicated that BPA exposure may be slightly declining whereas BPS exposure is increasing [9]. However, BPS and BPF are considered unsafe alternatives to BPA [10–13], highlighted by a recent review reporting BPS and BPF have similar potential endocrine-disrupting actions (high estrogenic and androgenic activity) as BPA [14].

Given the potential relationship between bisphenol exposure and adverse health outcomes, intervention studies targeting diet and education have tried to reduce exposures, with mixed results [15–17]. Sathyanarayana et al. observed that a 5-day randomized dietary replacement study actually increased urinary BPA [17], but the food provided to participants was likely contaminated with endocrine disruptors. In contrast, we showed that in women of normal weight, a 3-week intervention targeting BPA exposures from food, cosmetics, and other packaged products significantly (*P* < 0.05) reduced urinary BPA by 50% (− 1.06 ng/mL) whereas controls increased urinary BPA by 62% (+ 0.85 ng/mL), but BPS and BPF were not assessed [15]. Additionally, we noted a small, albeit significant difference in a 3-week weight (*P* = 0.03; − 0.28 kg weight loss vs. + 1.65 kg weight gain) in intervention vs. control participants, respectively, in our sample of normal-weight women; this might have been due to self-monitoring of caloric intake that occurred in the intervention group. These and other [16] data suggest that short-term interventions may successfully reduce BPA exposure in women with normal weight. Surprisingly, no published study to date has directly assessed whether a similar intervention can reduce bisphenol exposure in women with obesity. This is of concern because individuals with obesity may have higher urinary concentrations of bisphenols, and women in particular may be at the greatest risk [6, 18, 19]. Additionally, higher concentrations of BPA in women with obesity of reproductive age are associated with obesity, insulin resistance, and polycystic ovary syndrome and may lead to disruption of reproductive function [20, 21]. Thus, efficacious interventions to reduce bisphenol exposure in women with obesity are needed.

## Objectives

The primary objective of this study was to develop and test the feasibility of a 3-week behavioral change intervention, rooted in social cognitive theory [22, 23], to lower a broad range of bisphenols (BPA, BPS, and BPF) in women with obesity. The intervention was designed to targeted bisphenol exposures from food, personal hygiene products, cosmetics, and feminine hygiene products.

Secondary objectives were to examine weight changes and explored association with urinary bisphenols.

## Methods

### Participants

Thirty, healthy, premenopausal women with obesity from the California Polytechnic State University in San Luis Obispo, CA, were recruited on campus (Table 1) and assessed from November 2015 through November 2017. Eligibility included (1) female with obesity (> 30.0 kg/m^2^ BMI), (2) disease-free and non-smoking as assessed by a health history questionnaire, and (3) self-reported exposure to at least 5 potential dietary sources of bisphenols; for this, women completed a modified version of a 2-day diet practices survey to identify exposure to BPA as previously described [15, 16] and (4) self-reported daily use of at least 13 of 24 (> 50%) non-dietary product sources of BPA, including makeup products (foundation, mascara, eye shadow, etc.), hygiene products (hand sanitizer, face and body lotion, soap, shampoo, etc.), and feminine products (tampons, pads). This method was used successfully in our previous intervention trial where < 10% of participants at study entry had urinary BPA below the lowest detectable level [15]. The Institutional Review Board at California Polytechnic State University approved the study, and all women gave verbal and written consent. This study was carried out in accordance with The Code of Ethics of the World Medical Association (Declaration of Helsinki).

**Table 1:** Participant characteristics. Values are the mean (SD)

	Control	Intervention
Number of participants	15	15
Age (years)	21.5 (3.1)	21.5 (3.3)
Weight (kg)	81.5 (14.4)	84.1 (17.0)
Height (m)	1.6 (0.1)	1.7 (0.1)
Body mass index (kg/m^2^)	30.8 (5.8)	31.5 (5.6)
Hispanic/Latina, number, %		
Yes	3, 20%	2,13%
No	12, 80%	13, 87%

### Experimental protocol and design of intervention

After eligibility was determined, women reported to the Department of Kinesiology and Public Health at California Polytechnic State University in San Luis Obispo in the morning after an overnight fast (8–10 h). Research assistants blinded to treatment allocation completed all assessments. Weight was measured in duplicate at study entry and after 3 weeks in kilogram by a standard balance beam scale, and height by a stadiometer to the nearest centimeter. Women gave a fasting urine sample at study entry and after 3 weeks. The primary outcome measure was bisphenol (BPA, BPS, BPF) and creatinine concentrations, and the secondary outcome measure was weight.

This study adhered to the CONSORT guidelines for reporting randomized parallel pilot and feasibility studies [24]. At study entry, women were randomly assigned, by a computer-generated program, to the control group or the 3-week intervention group to decrease bisphenol exposure. The study statistician developed the randomization and provided concealed envelopes to study counselors with group assignments at study entry. The counselor did not know of the group assignment until the envelope was opened by the participant. Women in the control group received a weekly email newsletter that provided general information about bisphenol exposure, healthy eating, and beverages and did not receive any information about food or other packaging sources of bisphenol exposure. Women in the intervention group received all aspects of the control group, plus a behavioral intervention designed to decrease bisphenol exposure. The intervention was rooted in social cognitive theory [22, 23] and designed to reduce bisphenol exposure. The intervention promoted self-regulation skills (i.e., planning, self-monitoring, problem solving, goals, self-incentives) as well as positive reinforcement for adherence to behavior change goals and counselor feedback. The intervention included weekly face-to-face meetings with the counselor. At the first meeting, the counselor discussed the negative health consequences of bisphenols and how to avoid exposures through daily changes in diet and personal hygiene; strong emphasis was placed on the consumption of organic foods and tracking and changing intake of packaged foods. Women were encouraged to avoid canned/plastic containing food and beverages. Women were instructed to bring in their plastic containing products including Tupperware, dishware, cosmetics, and hygiene products (e.g., shampoo, condition, lotion), and these were returned after study completion. Participants were provided, free of charge, with replacement bisphenol-free glass Tupperware, water bottles, make-up, hygiene, and feminine products. The make-up, hygiene, and feminine products were packaged in BPA-free plastic containers, glass, and/or cardboard; this information was provided by the manufactures, and we did not directly test bisphenol levels in the packaging. Women were asked to self-monitor type (i.e., organic vs. non-organic) and packaging (plastic, glass, cardboard, other materials) of all food and beverages, but did not record quantity or caloric values of food. At the subsequent face-to-face visits, women were provided feedback on self-monitoring records and encouraged to continue self-monitoring and avoiding bisphenol containing products with the goal of all foods and drinks consumed packaged in bisphenol-free, glass, and cardboard containers or materials.

### Urinary analysis

Fifteen milliliters of fasting urine were collected mid-stream in a bisphenol-free sterile specimen container and then aliquoted into 3 separate bisphenol-free polypropylene tubes and stored at − 80 °C until analyzed. All urine samples were assessed in duplicate by gas chromatographic-tandem mass spectrometric (GC-MS/MS) method using isotope dilution quantification using the established CDC protocol [18] and using good laboratory practices as previously described [25, 26]. Bisphenols were not detected in urine collection tubes and storage apparatus. The limit of detection (LOD) was 0.05 μg/L for BPA, BPS, and BPF. Urinary creatinine concentrations were assessed by a colorimetric assay. Urine collection occurred from November 2015 to November 2017, and samples were analyzed in batches no more than 2 months after collection. Samples were sent under strict protocols (overnight shipping on dry ice, 2 unique identifiers, etc.) to the Washington State Public Health Laboratories (CLIA certified) for analysis of bisphenols.

### Statistical analyses

The sample size calculation for this study was based on our previous randomized controlled study [15] of 24 normal-weight women that showed that an intervention significantly reduced urinary BPA (− 1.06 ± 2.1 ng/mL) whereas controls increased urinary BPA (+ 0.85 ± 0.74 ng/mL). With 30 women at study entry and assuming 10% non-detectable BPA values at baseline, this study was projected to have > 99% power to detect a ≥ − 1.91 ng/mL difference in urine BPA concentrations between women in the intervention and control groups using a *α* = 0.05 and a 2-sided *t* test. Thus, recruitment was stopped at 30 participants. Based on NHANES [27] BPA median data of 2–3 ng/mL, a reduction of − 1.91 ng/mL in urine BPA is equivalent to > 35% decrease from the median.

A commercial software package from SPSS version 24 was used for statistical analysis of data. Summary statistics are reported as mean (SD) for participant characteristics; creatinine-corrected geometric mean, change in creatinine-corrected geometric mean, and 95% confidence interval for urinary BPA, BPS, and BPF concentrations; and mean ± SD for creatinine concentrations and body weight. Non-detectable BPA, BPS, and BPF concentrations were assigned the LOD (0.05 μg/L) [28]. Bisphenol concentrations were not normally distributed and were log-transformed prior to analysis. For secondary objectives, a RMANOVA was used to assess treatment × time effects on weight adjusting for study entry BMI and explored associations between weight and urinary bisphenol concentrations, adjusting for the group. An intent-to-treat approach (baseline data carried forward) was used for any missing data.

## Results

**Fig. 1:**
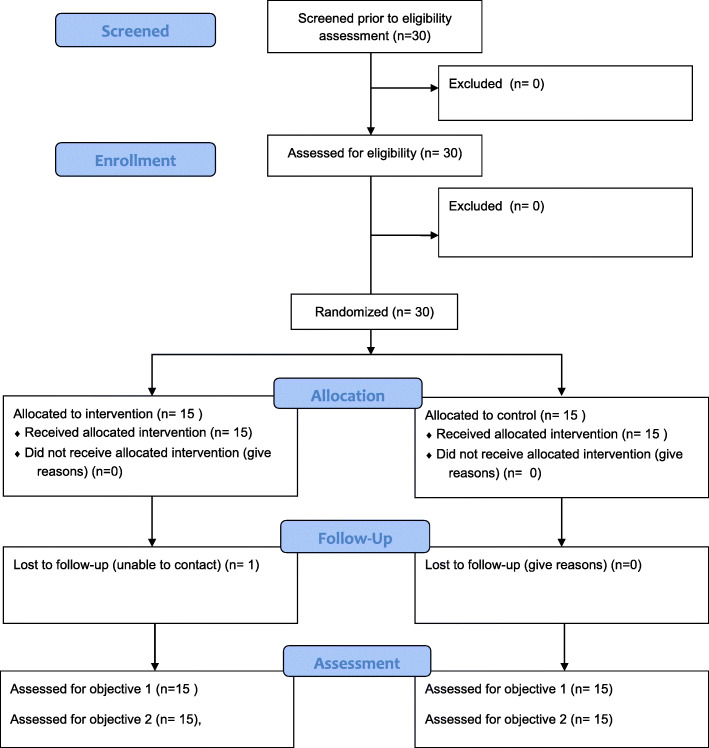
Flow diagram

Figure 1 shows the CONSORT flow diagram. Over the course of the study, all women expressing interest in the study were randomized; thus, enrollment and recruitment were 100%. Overall retention was 96%, as one intervention woman was lost to follow-up due to unable to contact, and no women in the control group were lost to follow-up. Adherence to the bisphenol avoidance intervention was estimated based on the completion of self-monitoring records over time. In the intervention group, the number of daily records completed was 7.7 ± 1.3 (mean ± SD) after week 1, 7.1 ± 1.5 after week 2, and 4.4 ± 0.9 after week 3. Based on CONSORT guidelines, there were no harms or unintended effects for each treatment.

Table 2 presents geometric mean and 95% confidence intervals for urinary creatinine-corrected BPA, BPS, and BPF concentrations. Non-detectable levels of bisphenols were observed but did not significantly vary by groups. At study entry, 15 of 30 BPA concentrations (50%) were non-detectable (7 intervention, 8 control), 1 of 30 BPS concentrations (0.04%) was non-detectable, and 13 of 30 BPF concentrations (47%) were non-detectable (6 intervention, 7 control). Among the remaining participants with detectable values, creatinine-corrected BPA and BPF did not significantly differ between groups but differences in BPS were observed, with higher creatinine-corrected BPS concentrations in intervention vs. controls.

**Table 2:** Geometric mean urinary creatinine-corrected BPA, BPS, and BPF concentrations and 95% confidence interval in control and intervention groups

	Control (***N*** = 15)		Intervention (***N*** = 15)	
	Study entry GM (95% CI)	3 weeks GM (95% CI)	Study entry GM (95% CI)	3 weeks GM (95% CI)
BPA, μg/g creatinine	0.19 (− 0.21, 1.27)	0.24 (0.08, 1.03)	0.32 (0.25,1.55)	0.32 (0.19, 1.15)
BPS, μg/g creatinine	0.73 (0.58, 2.34)	0.64 (0.86, 2.06)	2.31 (2.03, 3.81)	1.04 (0.69, 1.89)
BPF, μg/g creatinine	0.13 (− 4.55, 3.98)	0.12 (− 0.14, 2.41)	0.79 (1.73, 10.25)	0.24 (− 0.54, 2.01)

*BPA* bisphenol A, *BPS* bisphenol S, *BPF* bisphenol F, *GM* geometric mean, *CI* confidence interval

In the secondary analysis, using an intent-to-treat approach and adjusted repeated measures analyses, there was a significant treatment × time (*P* = 0.01) effect on creatinine-corrected urinary BPS concentrations. From study entry to 3 weeks, geometric mean creatinine-corrected urinary BPS concentrations significantly declined by 1.42 μg/g creatinine in the intervention group and were reduced slightly by 0.09 μg/g creatinine in the control group. There were no significant main or treatment × time effects on creatinine-corrected urinary BPA and BPF concentrations between groups. Our analysis assumed a LOD of 0.05 μg/L for each bisphenol, and in post hoc power analysis, the study was not sufficiently powered (32% power for both BPA and BPF) excluding the large amount of non-detectable. Completers in the intervention had − 0.31 μg/g creatinine and − 2.26 μg/g creatinine reduction in BPA and BPF vs. completers in the control had + 0.21 μg/g creatinine and − 0.80 μg/g creatinine in BPA and BPS, but there was no significant difference between groups. Examining urinary creatinine, there was no significant treatment × time effect observed from study entry to 3 weeks in the intervention (980 ± 778 and 1270 ± 891 mg/l, respectively) and control (1637 ± 954 and 1468 ± 1156 mg/l, respectively) groups.

There were no significant main or treatment × time effects on weight; the mean weight change in the intervention was 0.1 ± 1.0 kg and in the control was − 0.2 ± 1.2 kg. Changes in creatinine-corrected BPA, BPS, and BPF were not significantly associated with each other or associated with weight changes.

## Discussion

The primary objective of this study was to develop and test the feasibility of a 3-week behavioral intervention to reduce bisphenols. The intervention, rooted in social cognitive theory, included weekly face-to-face meetings to reduce bisphenol exposures from food, cosmetics, and packaged products. The intervention was evaluated as feasible. Recruitment and enrollment were 100%, and overall retention was 96% as one intervention woman was lost to follow-up and no women in the control group were lost to follow-up. Adherence to the bisphenol avoidance intervention, estimated based on completion of self-monitoring records, was high early on and decreased over-time. In the intervention group, the number of daily records completed on average was 7.7 after week 1, 7.1 after week 2, and 4.4 after week 3. Based on these feasibility results, a large scale, clinical trial intervention to reduce bisphenol exposure is warranted.

In the secondary analysis, the 3-week behavioral intervention reduced BPS exposure relative to controls in women with obesity. Geometric mean urinary creatinine-corrected BPS declined by 1.42 μg/g creatinine in the intervention and slightly decreased by 0.09 μg/g creatinine in controls, underscoring the short-term ability of an intervention to decrease urinary bisphenols in college-aged women with obesity. These data need to be interpreted with caution given the relatively small sample size in each group, and the large number of non-detectable BPA and BPF concentrations in both groups.

Prior studies have examined interventions to reduce bisphenol exposure in women of normal weight. One non-randomized study found significant reductions in BPA and phthalates after a 3-day “fresh food” diet [16]. Another 5-day randomized dietary replacement study showed an actual increase in BPA [17], but the food provided to the intervention group was DEHP contaminated and possibly also had BPA contamination. Our previous randomized controlled study showed that a similar 3-week intervention successfully reduced BPA concentrations in women of normal weight, but BPS and BPF were not assessed [15]. Surprisingly, no published study to date has assessed whether a similar intervention may lower bisphenols in women with obesity. The current study adds to the literature suggesting that an intervention may potentially impact BPS in women with obesity.

In the current study, we chose to enroll women with obesity as preliminary evidence suggests that this population is typically exposed to higher bisphenol concentrations compared to women of normal-weight and men [4]. Higher BPA exposure in women with obesity of reproductive age is associated with obesity, insulin resistance, and polycystic ovary syndrome and may lead to disruption of reproductive function [20, 21]. However, the exact mechanism(s) of higher exposure in women with obesity is unclear, although diet and acquisition of canned foods are primary exposure to bisphenols [29]. Future intervention studies are needed to determine whether a similar intervention would be beneficial to others including men, and at-risk groups including diabetics.

BPS and BPF, analogs of BPA with similar chemical structures, are increasingly used as substitutes for BPA in packaging and industry products. In particular, BPS is a stronger acid and more stable than BPA, allowing it to be more resistant to heat and sunlight [30]. Studies have shown that BPS is present in food packaging and foodstuff [31], paper products [32], and a variety of personal care products including body wash, hair care, skin lotion, and shampoo [33]. A recent observational study indicated that BPA exposure in the USA may be slightly declining whereas BPS exposure is increasing [9]. However, BPS and BPF are considered unsafe alternatives to BPA [10–13], highlighted by a current review reporting BPS and BPF have endocrine-disrupting actions [14]. In particular, animal data suggest that BPS has estrogenic activity, impairs blood functioning and increases cardiovascular risk, and has a stronger effect on reducing testosterone concentrations than BPA [14, 34, 35]. Thus, interventions to reduce a broad range of bisphenols, and not necessarily just BPA, are needed.

We observed no significant intervention changes in BPA and BPF concentrations, which likely reflect the high rate of baseline non-detectable levels of these bisphenols. At baseline, 50% and 47% of participants had non-detectable BPA and BPF, respectively, which is higher than previous observational studies [18]. In post hoc power analysis, this study was insufficiently powered to detect changes in BPA and BPF due to the large non-detects. Unlike the current study, in our previous intervention study in women with normal weight, < 10% of all baseline samples had non-detectable BPA and geometric mean baseline samples were similar to the reference NHANES data [27]. In the current study in women with obesity, the adipose tissue may be acting as a bisphenol storage site, thereby removing bisphenols from circulation (and ultimately urinary excretion), particularly in individuals with obesity [36]. Alternatively, the large non-detectable could reflect a decline in exposure to these bisphenols occurring nationally [9]. To assess bisphenol exposure prior to randomization, we used a survey from prior studies [15, 16] and future research should consider directly assessing urinary bisphenol concentrations during screening to ensure baseline exposure consistent with the general population prior to randomization.

In secondary analyses, the bisphenol intervention had no impact on weight changes, and there was no relationship between changes in weight and bisphenol concentrations. Previously, we showed that a BPA intervention, compared to controls, significantly reduced a 3-week weight gain (− 0.28 kg weight loss vs. 1.65 kg weight gain) [15]. In an effort to increase awareness of BPA-containing food packaging, our previous intervention encouraged self-monitoring of food and calorie intake with daily diaries [37], and this may have unintentionally led to weight changes between groups. In the current study, we modified the intervention and instructed women to record food packing only and women did not record calories; we observed no significant weight changes. However, it is important to note that the current study did not control for many diet variables, which potentially confounds the relationship between bisphenols and weight. Future research is needed to untangle the relationships between bisphenol exposure, dietary changes, and weight status.

There are notable strengths and limitations of the current study. We experimentally tested the feasibility, using a randomized controlled trial consistent with CONSORT guidelines [38], of an intervention to reduce a broad range of bisphenol exposures that included promoting healthy organic foods, and daily self-monitoring of bisphenol exposures and provided product labeled BPA-free in women with obesity. Despite the success of the intervention to reduce BPS exposure, results should be interpreted with caution given the relatively small sample size of this study and the large number of non-detectable BPA and BPF concentrations. Only spot urine samples were collected, which may have been insufficient to reliably estimate a long-term urinary bisphenol exposure as a recent study suggested that multiple samples over several days are needed [39]. Previous studies have shown that urinary BPA concentrations vary daily [40] and may be influenced by recent food intake [41]. To minimize these potential confounds, we took great care in collecting urine samples and used good laboratory practices in our analyses (e.g., assessment of blanks, collection, and storage tubes, etc.). Also, randomization led to higher BPS exposure in the intervention group vs. control, although this was adjusted for statistically. We did not assess whether intervention reductions in bisphenols were related to improvements in cardiometabolic health risk markers (e.g., glucose, insulin, lipid profile). We did not assess the intervention on other non-persistent endocrine-disrupting chemicals with known negative health consequences (e.g., phthalates) [42, 43]. Finally, we recruited a convenient sample of college-aged women with obesity, and it is unclear whether the intervention would have the ability to reduce bisphenol exposure in men and other at-risk groups including diabetics.

## Conclusion

In conclusion, the primary objective of this study is to develop and test the feasibility of a 3-week behavioral change intervention to lower bisphenols were met. Specifically, we developed a novel behavioral intervention, rooted in social cognitive theory, with high retention and adherence in college-aged women with obesity. A future large-scale clinical randomized trial in women is currently in development collecting repeat urine samples over multiple days, assessing a broad range of endocrine-disrupting chemicals, and determining whether reducing exposures to endocrine-disrupting chemicals has a positive impact on health outcomes related to the pathogenesis of cardiovascular disease and type 2 diabetes.

## Data Availability

Datasets used and/or analyzed during the current study are freely available from the California Polytechnic State University Digital Commons repository (https://digitalcommons.calpoly.edu/kine_fac/133/).
